# VDR Polymorphisms in Autoimmune Connective Tissue Diseases: Focus on Italian Population

**DOI:** 10.1155/2021/5812136

**Published:** 2021-12-23

**Authors:** Andrea Latini, Giada De Benedittis, Carlo Perricone, Serena Colafrancesco, Paola Conigliaro, Fulvia Ceccarelli, Maria Sole Chimenti, Lucia Novelli, Roberta Priori, Fabrizio Conti, Cinzia Ciccacci, Paola Borgiani

**Affiliations:** ^1^Department of Biomedicine and Prevention, Genetics Section, University of Rome Tor Vergata, 00133 Rome, Italy; ^2^Rheumatology, Department of Medicine, University of Perugia, Piazzale Giorgio Menghini, 1, 06129 Perugia, Italy; ^3^Division of Rheumatology, Department of Clinical Internal, Anaesthesiologic and Cardiovascular Sciences, Sapienza University, 00133 Rome, Italy; ^4^Rheumatology, Allergology and Clinical Immunology, Department of “Medicina dei Sistemi”, University of Rome Tor Vergata, 00133 Rome, Italy; ^5^UniCamillus, Saint Camillus International University of Health Sciences, 00131 Rome, Italy

## Abstract

Vitamin D is an important hormone involved in various physiologic processes, and its activity is linked to binding with vitamin D receptor (VDR). Genetic polymorphisms in the VDR gene could modulate the expression or function of the receptor and, consequently, alter the effects of vitamin D. Variants in VDR gene have been associated with susceptibility to many illnesses sensitive to vitamin D administration and to autoimmune disorders, but no data are available regarding autoimmune connective tissue diseases in Italian population. We analyzed three VDR polymorphisms in 695 Italian patients with autoimmune connective tissue diseases (308 with systemic lupus erythematosus (SLE), 195 with primary Sjogren's syndrome (pSS), and 192 with rheumatoid arthritis (RA)) and in 246 healthy controls with the aim to evaluate a possible association of VDR SNPs with susceptibility to these diseases in the Italian population. Genotyping of rs2228570, rs7975232, and rs731236 in VDR gene was performed by an allelic discrimination assay. A case/control association study and a genotype/phenotype correlation analysis have been performed. We observed a higher risk to develop SLE for rs2228570 TT genotype (*P* = 0.029, OR = 1.79). No association was observed between susceptibility to pSS or RA and this SNP, although this variant is significantly less present in RA patients producing autoantibodies. For rs7975232 SNP, we observed a significant association of the variant homozygous genotype with SLE (*P* = 0.009, OR = 1.82), pSS (*P* = 0.046, OR = 1.66), and RA (*P* = 0.028, OR = 1.75) susceptibility. Moreover, we reported associations of this genotype with clinical phenotypes of SLE and pSS. Lastly, the GG genotype of rs731236 was associated with a lower RA susceptibility (*P* = 0.045, OR = 0.55). Our results show that the explored VDR polymorphisms are significantly associated with autoimmune connective tissue disorders and support the hypothesis that the genetic variability of VDR gene may be involved in susceptibility to these diseases in Italian population.

## 1. Introduction

Vitamin D is a pleiotropic hormone, which is involved in various physiologic processes, such as phosphate and calcium homeostasis, cell growth, and neuromuscular and immune function [1]. The biological activity of vitamin D is closely linked to the binding with the vitamin D receptor (VDR), a member of the nuclear receptor family of transcription factors. The active form of vitamin D, calcitriol (1*α*,25(OH)_2_D_3_), binds to VDR, enters in the nucleus, and recognizes the promoter regions known as vitamin D responsive element (VDRE), activating the expression of specific gene products [2]. Variations in the activity of VDR or in its affinity with the ligand can significantly alter the functions of vitamin D. Genetic polymorphisms in the VDR gene could modulate the expression or function of the receptor and consequently could be involved in the variability of the vitamin D effects [3].

Genetic variability in the VDR gene has been investigated in numerous human diseases sensitive to vitamin D administration, and the effects of various polymorphisms have been evaluated [4]. VDR SNPs (single nucleotide polymorphisms) have been also described as associated to autoimmune diseases, such as systemic lupus erythematosus (SLE) [5], rheumatoid arthritis (RA) [6], or multiple sclerosis (MS) [7]. Vitamin D, in fact, regulates the maturation of various immune cell lines, such as dendritic cells, monocytes, and activated T and B cells. These cells express VDR gene and respond to the active form of vitamin D [8]. Low levels of calcitriol have been associated with an increase in proinflammatory mediators and reported in several autoimmune diseases, such as MS, SLE, and diabetes mellitus [9]. However, significant discrepancies have emerged from the findings of association studies, highlighting how the associations between VDR polymorphisms, and the different diseases vary considerably in different ethnic groups, even within the same geographical area [10]. Previous studies have shown a positive association between VDR gene polymorphisms and various diseases in the Italian population, for example, breast cancer [11], osteoporosis [12], or periodontitis [13]. Although the role of vitamin D in the immune system is well known [14], genetic association studies of VDR SNPs on autoimmune connective tissue diseases in Italian population are lacking.

At least 14 different polymorphisms with possible functional effect have been described in the VDR gene, of which FokI (rs2228570), ApaI (rs7975232), and TaqI (rs731236) are probably the most studied [14]. In fact, independent correlations of these polymorphisms with the modulation of vitamin D status have been described, both in original articles and in meta-analyses [14].

Since the associations among autoimmune connective tissue diseases and VDR gene polymorphisms have been described in many populations, in the present study, we aimed to investigate whether these associations could be confirmed also in Italian population. We analyzed the distribution of three VDR polymorphisms in three different autoimmune connective tissue disorders, specifically systemic lupus erythematosus (SLE), primary Sjogren syndrome (pSS), and rheumatoid arthritis (RA), in Italian patients and in healthy controls (HCs), with the aim to evaluate their possible association with the disease susceptibility. Furthermore, we evaluated the possible involvement of these polymorphisms in different clinical manifestations of the three diseases.

## 2. Materials and Methods

### 2.1. Sample Collection

We enrolled 695 subjects with autoimmune connective tissue disorders, comprehending 308 SLE patients, 195 pSS patients and 192 RA patients, and 246 HCs. SLE patients, which diagnoses were made according to the revised 1997 American College of Rheumatology Criteria, were recruited from 2012 to 2016 at the Lupus Clinic of the Rheumatology Unit (Sapienza, University of Rome). The pSS patients were diagnosed according to the American European Consensus Criteria and enrolled from 2015 to 2016 at Sjӧgren's Clinic of the Rheumatology Unit (Sapienza, University of Rome). RA patients were diagnosed according to the 2010 American College of Rheumatology (ACR)/European League Against Rheumatism (EULAR) classification criteria. They were recruited from 2014 to 2016 at the Rheumatology Outpatient Clinic at the Department of “Medicina dei Sistemi” (Policlinico Tor Vergata, Rome, Italy). Demographic and clinical description of patients included in this study was previously reported [15–18]. A written informed consent was signed by all patients. The study protocol was approved by the local ethics committee of the Sapienza University of Rome for SLE and pSS samples and University of Rome “Tor Vergata” for RA samples. Peripheral blood samples from all patients and controls have been collected and stored at -20°C until usage.

### 2.2. DNA Extraction and Genotyping

Genomic DNA was isolated from whole blood using a Qiagen blood DNA mini kit (Qiagen, Valencia, VA, USA) with standard procedure. Patients and HCs were analyzed for three polymorphisms in VDR gene (rs2228570, rs7975232, and rs731236) by an allelic discrimination assay (Applied Biosystems, Foster City, CA, USA) and 7500 real-time instrument (Applied Biosystems-Thermo Fisher, CA, USA). In each run of the allelic discrimination assay, we used samples with known genotypes, confirmed by direct sequencing, as genotype controls.

### 2.3. Statistical Analysis

Pearson's *χ*2 test has been used to verify the Hardy–Weinberg equilibrium for all SNPs and to compare genotype frequencies of patients with respect to those of HCs. Odds ratios (ORs) with 95% CI were calculated. A genotype-phenotype correlation analysis has been performed to compare cases with and without specific manifestations. Haploview 4.2 has been used to infer the haplotypes between the three VDR SNPs. Chi-square test has been applied to evaluate the differences in haplotype distribution between cases and HCs. Two-tailed *P* values less than 0.05 were considered statistically significant. Statistical analyses were performed by the SPSS program ver. 19 (IBM Corp., Armonk, NY, USA).

## 3. Results

In this study, we enrolled 308 SLE patients, 195 pSS patients, 192 RA patients, and 246 HCs. Hardy-Weinberg equilibrium in healthy controls group has been verified for each investigated SNPs. The distributions of genotype frequencies of three VDR polymorphisms (rs2228570, rs7975232, and rs731236) in the patients were compared with those in healthy controls. Among the different models performed, the recessive model showed the most significant associations. The VDR genotype distribution and the case/control association analysis are reported in Table 1.

As shown, the variant homozygous genotype (TT) of rs2228570 SNP was associated with SLE susceptibility (*P* = 0.029 and OR = 1.79). Conversely, no statistically significant associations were observed for pSS and RA.

Regarding the rs7975232 SNP, interestingly, the variant homozygous genotype (GG) was associated with the susceptibility to all investigated autoimmune connective tissue disorders. Indeed, individuals homozygous for the rs7975232 variant allele showed a higher risk of developing SLE (*P* = 0.009, OR = 1.82), pSS (*P* = 0.046, OR = 1.66), and RA (*P* = 0.028, OR = 1.75), respectively.

Lastly, rs731236 SNP was associated with RA susceptibility. The GG genotype was in fact significantly less prevalent in RA patients than in controls (*P* = 0.045, OR = 0.55), so it seems to decrease the risk to develop the disease.

For each disease, haplotype distributions among the three SNPs were inferred and compared to HC haplotype frequencies. The haplotype analyses did not improve the statistical significance with respect to the single SNP associations (data not shown).

A genotype/phenotype correlation analysis between VDR polymorphisms and patients clinical phenotypes was carried out (Table 2). The rs2228570 TT genotype seemed to confer a protective effect with respect to the reduction of C4 (*P* = 0.003, OR = 0.36) and C3 (*P* = 0.021, OR = 0.45) levels in SLE patients. Interestingly, it was also significantly less present in RA patients producing autoantibodies (*P* = 0.034, OR = 0.33).

The rs7975232 GG genotype seems to be involved in predisposition to produce autoantibodies, in SLE and pSS patients. Indeed, this SNP was associated with production of anti-DNA (*P* = 0.024, OR = 0.52), anti-RNP (*P* = 0.05, OR = 1.97), anti-SSA (*P* = 0.035, OR = 1.87), and anti-SSB (*P* = 0.037, OR = 2.16) autoantibodies, in SLE patients, and with production of anti-SSA (*P* = 0.033, OR = 0.47) in pSS. Moreover, it was associated with a higher risk to develop neuropsychiatric lupus symptoms (*P* = 0.023, OR = 2.24).

Although the rs731236 polymorphism is not directly associated with the susceptibility to SLE and pSS, it seems to modulate the variability of clinical manifestations in these two diseases. In SLE patients, the GG genotype showed a protective effect with respect to develop neuropsychiatric symptoms (*P* = 0.037, OR = 0.24), leukopenia (*P* = 0.024, OR = 0.49), and nephritis (*P* = 0.032, OR = 0.042) but also a higher predisposition to produce anti-SSB autoantibodies (*P* = 0.027, OR = 2.41). Moreover, pSS patients carrying this genotype have a higher risk to develop parotid swelling (*P* = 0.014, OR = 2.84).

## 4. Discussion

Vitamin D deficiency was frequently observed in autoimmune diseases patients. Variability in vitamin D effects could be partly explained by polymorphisms located in genes involved in its metabolism, including VDR. Both the levels of vitamin D active form and genetic variants that influence its expression or function could contribute, albeit modestly, to the predisposition to autoimmune diseases with complex aetiology, such as the three diseases considered in this study.

Among the most common VDR polymorphisms, rs2228570 (FokI) is a missense polymorphic variant, located in exon 2, which causes a change of threonine to methionine. The variant allele introduces a new start codon, which generates 3 amino acids shorter VDR protein compared to full-length VDR isoform of 427 amino acids [19]. Rs7975232 (ApaI) SNP is instead located in the intron 8 and could affect mRNA stability and the gene expression of VDR [20]. Rs731236 (TaqI) is a synonym variant located in the exon 9 and not affects the amino acid sequence. The variant allele of this polymorphism could have an effect on mRNA stability or modify the binding with VDREs located in the target genes [21].

In recent years, numerous studies have investigated the association of VDR gene polymorphisms with the risk of developing autoimmune diseases throughout different populations. Nonetheless, these studies reported conflicting results [14]. Discrepancies among association studies may be due to small sample sizes, low statistical power, clinical heterogeneity of the patients, variations in the disease diagnosis, but mainly to different allelic distributions among different populations. Of course, different genetic factors could interact with environmental risk factors existing in different geographic regions, modulating differences in the population susceptibility to specific diseases. Therefore, in this study, we explored a possible role of common SNPs of VDR gene in Italian patients affected by three autoimmune connective tissue disorders, each one represented by a clinically well characterized cohort of patients. Indeed, although there are numerous studies and meta-analysis on VDR SNPs in many diseases, these never analyzed the Italian patients with autoimmune connective diseases.

Our analyses indicated that the explored VDR polymorphisms were significantly associated with these diseases also in Italian population. In particular, the rs2228570 was associated with SLE susceptibility, and the rs731236 was associated with RA susceptibility, while rs7975232 was associated with all three diseases. Specifically, variant genotypes of rs2228570 and rs7975232 are associated with an increased risk of developing the disease, while rs731236 seems to have a protective effect.

Functional studies showed that the short VDR isoform is 1.7-fold more active than the long VDR isoform, both in terms of its transactivation capacity as a transcription factor [3].

The TT genotype of rs2228570 was associated with SLE susceptibility in different meta-analyses [22–24], in particular in the Arab and Asian populations, and it was correlated with an higher SLEDAI score (Systemic Lupus Erythematosus Disease Activity Index) [25]. We also observed a significant association with SLE susceptibility, so we suggest that this polymorphism could contribute to SLE risk also in our population.

Variant allele of rs2228570 was associated with RA susceptibility in three meta-analyses on different populations [26–28], but we could not confirm this association in our cohort. A recent study evaluated the distribution of VDR polymorphisms in Italian patients affected from juvenile idiopathic arthritis versus healthy controls, and no significant difference was found concerning the rs2228570 [29]. We can therefore hypothesize that this polymorphism does not contribute to the risk of developing arthritis in the Italian population.

In our study, we observed that individuals homozygous for the rs7975232 variant allele showed a higher risk of developing all investigated autoimmune connective tissue disorders. Data in literature concerning SLE or RA susceptibility reported a wide heterogeneity of results. Indeed, many studies did not find an association between rs7975232 and risk of developing SLE [24] or RA [28], while other studies reported a different distribution of this SNP between patients and healthy controls [30, 31]. However, none of these studies investigated the Italian population. We showed that rs7975232 seems to associate also with pSS. To our knowledge, there is only one study about the VDR gene polymorphisms in pSS. In fact, VDR polymorphisms were analyzed in Hungarian pSS patients, but no association was observed, not even with the rs7975232 polymorphism [32]. Therefore, the relationship of rs7975232 with these autoimmune diseases does not seem to be clear and could be strongly influenced also by ethnicity.

The majority of meta-analyses showed no significant association of rs731236 to the SLE risk [22–24], in accordance with our study. On the contrary, we observed that the variant genotype of rs731236 was significantly less prevalent in RA patients than in controls. The variant homozygous genotype of rs731236 was associated to RA with a protective effect also in a meta-analysis in the African and Arab population [6].

Admittedly, our study presents some limitations: first to all, the relatively small number of patients for each disease and the lack of a replication cohort. However, our results appear promising and support the literature data on the association between VDR polymorphism and susceptibility to autoimmune connective tissue disorders.

## 5. Conclusion

In our study, we tried to increase the data already present in the literature, providing an analysis of VDR polymorphisms in autoimmune connective tissue diseases in the Italian population. The strength of this study is the very well clinically determined definition of the disease phenotypes and the diagnosis of each patient that was carried out in an accurate and homogeneous way, according to the current guidelines. Moreover, a scrupulous clinical characterization allowed highlighting associations of VDR polymorphisms with specific phenotypes of each disease, not always possible in GWAS or multicentre studies. Despite the significant associations reported, the present study should be replicated in a larger cohort. Moreover, some functional studies could also clarify the molecular mechanisms of these associations. Anyway, these data support the hypothesis that the genetic variability of VDR may be involved in susceptibility to autoimmune connective tissue disorders also in Italian population.

## Figures and Tables

**Table 1:** Genotype distribution of VDR polymorphisms in SLE, pSS, RA, and HCs.

rs2228570 C > T Fok1	TOT	CC	CT	TT	*P* ^a^	OR (95% CI)
HCs	246	103	120	23		
SLE	308	121	139	48	*0.029*	1.79 (1.05-3.05)
pSS	195	88	83	24	0.32	1.36 (0.74-1.49)
RA	192	87	87	18	1	1 (0.52-1.92)
rs7975232 T > G Apa1	TOT	TT	TG	GG	*P* ^a^	OR (95% CI)
HCs	239	82	124	33		
SLE	301	104	129	68	*0.009*	1.82 (1.15-2.87)
pSS	195	55	99	41	*0.046*	1.66 (1.01-2.75)
RA	192	52	98	42	*0.028*	1.75 (1.06-2.89)
rs731236 A > G Taq1	TOT	AA	AG	GG	*P* ^a^	OR (95% CI)
HCs	242	75	127	40		
SLE	300	113	138	49	1	0.99 (0.62-1.56)
pSS	195	74	94	27	0.44	0.81 (1.48-1.38)
RA	192	80	93	19	*0.045*	0.55 (0.31-0.99)

^a^ *P* value represents the analysis based on the recessive model (homozygous variant genotypes vs. others). HCs: healthy controls; SLE: systemic lupus erythematosus; pSS: primary Sjogren's syndrome; RA: rheumatoid arthritis; OR: odds ratio; CI: confident interval. Significant associations are reported in italic.

**Table 2:** Association between disease phenotypes and studied polymorphisms.

Disease	Phenotype	*P* ^a^	OR (95% CI)
rs2228570 C > T Fok1			
Systemic lupus erythematosus	C3 (below normal values)	0.003	0.36 (0.18-0.71)
	C4 (below normal values)	0.021	0.45 (0.23-0.89)
Rheumatoid arthritis	Seropositivity	0.034	0.33 (0.12-0.96)

rs7975232 T > G Apa1			
Systemic lupus erythematosus	Neuropsychiatric SLE	0.023	2.24 (1.11-4.54)
	Anti-dsDNA	0.024	0.52 (0.30-0.92)
	Anti-RNP	0.05	1.97 (0.99-3.92)
	Anti-Ro/SSA	0.035	1.87 (1.04-3.38)
	Anti-La/SSB	0.037	2.16 (1.04-4.50)
Primary Sjogren syndrome	Anti-Ro/SSA	0.033	0.47 (0.23-0.95)
	Seropositivity	0.043	0.49 (0.24-0.99)

rs731236 A > G Taq1			
Systemic lupus erythematosus	Leucopenia	0.024	0.49 (0.26-0.92)
	Nephritis	0.032	0.42 (0.19-0.95)
	Neuropsychiatric SLE	0.037	0.24 (0.06-1.02)
	Anti-La/SSB	0.027	2.41 (1.09-5.33)
	Seropositivity	0.045	1.96 (1.01-3.81)

Primary Sjogren syndrome	Parotid swelling	0.014	2.84 (1.20-6.70)

^a^ *P* value refers to the analysis based on the recessive model (homozygous variant genotypes vs. others).

## Data Availability

Genotyping data are available upon request.
